# Improving occupational physicians’ adherence to a practice guideline: feasibility and impact of a tailored implementation strategy

**DOI:** 10.1186/s12909-015-0364-8

**Published:** 2015-04-24

**Authors:** Margot CW Joosen, Karlijn M van Beurden, Berend Terluin, Jaap van Weeghel, Evelien PM Brouwers, Jac JL van der Klink

**Affiliations:** 1 Tilburg University, Tilburg School of Social and Behavioral Sciences, Tranzo Scientific Center for Care and Welfare, PO Box 90153, 5000 LE Tilburg, The Netherlands; 2 Department of General Practice and Elderly Care Medicine, VU University Medical Center Amsterdam, EMGO Institute for Health and Care Research, PO Box 7057, 1007 MB Amsterdam, The Netherlands; 3 Phrenos Centre of Expertise, PO Box 1203, 3500 BE Utrecht, The Netherlands; 4 Parnassia Group, Dijk en Duin Mental Health Center, PO Box 305, 1900 AH Castricum, The Netherlands; 5 Department of Health Sciences, Division of Community and Occupational Medicine, University Medical Center Groningen, University of Groningen, A. Deusinglaan 1, 9713 AV Groningen, The Netherlands

**Keywords:** Healthcare quality improvement, Implementation strategy, Perceived barriers, Mental health, Plan-do-check-act, Guideline adherence, Continuing medical education

## Abstract

**Background:**

Although practice guidelines are important tools to improve quality of care, implementation remains challenging. To improve adherence to an evidence-based guideline for the management of mental health problems, we developed a tailored implementation strategy targeting barriers perceived by occupational physicians (OPs). Feasibility and impact on OPs’ barriers were evaluated.

**Methods:**

OPs received 8 training-sessions in small peer-learning groups, aimed at discussing the content of the guideline and their perceived barriers to adhere to guideline recommendations; finding solutions to overcome these barriers; and implementing solutions in practice. The training had a plan-do-check-act (PDCA) structure and was guided by a trainer. Protocol compliance and OPs’ experiences were qualitatively and quantitatively assessed. Using a questionnaire, impact on knowledge, attitude, and external barriers to guideline adherence was investigated before and after the training.

**Results:**

The training protocol was successfully conducted; guideline recommendations and related barriers were discussed with peers, (innovative) solutions were found and implemented in practice. The participating 32 OPs were divided into 6 groups and all OPs attended 8 sessions. Of the OPs, 90% agreed that the peer-learning groups and the meetings spread over one year were highly effective training components. Significant improvements (p < .05) were found in knowledge, self-efficacy, motivation to use the guideline and its applicability to individual patients. After the training, OPs did not perceive any barriers related to knowledge and self-efficacy. Perceived adherence increased from 48.8% to 96.8% (p < .01).

**Conclusions:**

The results imply that an implementation strategy focusing on perceived barriers and tailor-made implementation interventions is a feasible method to enhance guideline adherence. Moreover, the strategy contributed to OPs’ knowledge, attitudes, and skills in using the guideline. As a generic approach to overcome barriers perceived in specific situations, this strategy provides a useful method to guideline implementation for other health care professionals too.

**Electronic supplementary material:**

The online version of this article (doi:10.1186/s12909-015-0364-8) contains supplementary material, which is available to authorized users.

## Background

Many evidence-based practice guidelines exist in health care, but adherence to these guidelines is generally low among care professionals [1-3]. Lack of adherence to practice guidelines can lead to omission of necessary care and contribute to preventable harm, suboptimal patient outcomes, or poor resources utilization [3]. Thus, implementation of and adherence to practice guidelines is important for improving the quality of patient care, and can also help decrease variability in treatment.

Various models have been developed which demonstrate that guideline implementation can be influenced by multiple factors, such as patient and practitioner characteristics, guideline and environmental factors, and the social-political context [4,5]. Accordingly, strategies to facilitate guideline implementation can have different orientation, such as professional-oriented, financial, organizational, and regulatory interventions. Although conclusive evidence of the effectiveness of implementation strategies is lacking [6-9], it is recognized that passive strategies such as guideline dissemination by itself are ineffective, and more active strategies are needed to improve guideline adherence [10,11]. Preferably, active implementation strategies should aim to eliminate barriers that hinder professionals from adhering to a specific guideline [12]. Cabana et al. [13] have shown that barriers to adherence can be knowledge-related such as a lack of awareness or familiarity, or attitude-related such as a lack of agreement, outcome expectancy, self-efficacy, or motivation. External barriers such as patient factors, guideline factors, and environmental factors may also play a role. In order to enhance implementation, perceived barriers should be analyzed for specific guideline recommendations, target group, and setting [14]. Subsequently, implementation interventions should be developed that are tailored to professionals’ needs to overcome the perceived barriers [14,15].

Although these tailored interventions seem promising, in practice the choice of an intervention is often not based on the identified barriers of the professionals but on researchers’ and implementers’ preferences or familiarity with specific interventions [16,17]. To avoid a mismatch between identified barriers and interventions, the target users of the guidelines should be actively involved in selecting the interventions that will overcome the barriers they encounter in practice. The successful removal of barriers through tailor-made interventions remains a black box phenomenon [16].

Evidence-based guidelines for occupational health professionals on the management of mental health problems (MHP) have been developed in various countries [18], however implementation into practice is challenging. Currently, MHP are among the leading causes of (work) disability worldwide [19], and can negatively impact work capacity and lead to sick leave and long-lasting work disability [20]. To address work disability due to MHP, The Netherlands Society of Occupational Medicine (NVAB) developed a practice guideline entitled ‘The management of mental health problems of workers by occupational physicians (OPs)’ in 2000 and revised it in 2007 [21,22]. In the Netherlands, the OP plays an important role in the return to work process of sick listed workers by assessing the worker’s work ability, giving advice about return to work and proving occupational health care. The NVAB guideline on mental health problems, referred to hereafter as ‘the MHP guideline’, promotes an activating approach by the OP aimed to establish earlier return to work and lower recurrence rates of workers on sick leave due to MHP (see Table 1). The guideline was distributed among Dutch OPs, and became part of their continuing medical education (nationally and locally) which enabled OPs to increase their knowledge of the guideline content. Subsequent research has shown that closer adherence to the guideline was associated with shortened sick leave duration in workers with adjustment disorders [23,24]. Although Dutch OPs had a positive attitude toward the guideline and intended to use it, actual compliance with the recommendations was limited [23,25].

**Table 1:** **Background information about the content of the ‘Mental Health Problems’ guideline** [22]

**1) Problem Orientation and Diagnosis**	An early involvement of the OP is promoted (first consultation about 2 weeks after the worker reports sick). A simplified classification of MHP is introduced in four categories: i) Stress-related complaints, ii) depression, iii) anxiety disorder, and iv) other psychiatric disorders. Furthermore, problem inventory should focus on factors related to the worker, his or her work environment, and the interaction between these two.
**2) Intervention/Treatment**	The OP acts as the case manager by monitoring and evaluating the process of recovery (process-based evaluation). If the recovery process stagnates, the OP should intervene by acting as the care manager by using cognitive behavioral techniques to enhance the problem-solving capacity of the worker, providing the worker and work environment with information/advice on the recovery and the RTW process, contacting the general practitioner if problems remain the same or increase, and referring the worker to a specialized intervention if necessary. In addition, the OP should advise the work environment (e.g., supervisors, managers, and human resource managers) on how to support the worker and enhance the recovery and RTW process.
**3) Relapse Prevention**	The integration of relapse prevention from the first contact with the worker is achieved by enhancing the problem-solving capacity of the worker.
**4) Evaluation**	During follow-up meetings, evaluation of the recovery process includes the perspectives of the worker, supervisor, and other involved professionals. Follow-up meetings with the worker should take place every 3 weeks during the first 3 months, and then every 6 weeks thereafter. The supervisor or work environment should be contacted once a month. Follow-up contacts with the general practitioner or other professionals should take place if the recovery process stagnates or if there is doubt about the diagnosis or treatment.

OP = occupational physician; MHP = mental health problems; RTW = return-to-work.

To improve adherence to the Dutch MHP guideline, we developed an implementation strategy to specifically target knowledge, attitude, and perceived external barriers, and to find solutions to overcome these barriers. OPs were actively involved in the identification of barriers and the implementation of solutions through the use of a Plan-Do-Check-Act (PDCA) approach in small-group interactive training meetings [26,27]. The objective of this article is to describe how this tailored implementation strategy for the MHP guideline was carried out, and to discuss how the strategy was received among the OPs. The following research questions were addressed:

1. How feasible is the tailored implementation strategy for the ‘Mental Health Problems’ guideline? Is the strategy carried out as planned, and what are the experiences of the target users of the guideline (i.e., the occupational physicians)?
2. What is the impact of the implementation strategy on occupational physicians’ knowledge, attitude, and perceived external barriers with regard to the guideline?

## Methods

### Implementation strategy

Based on scientific literature on the effectiveness of implementation strategies [11,15,28], we developed a (tailored) guideline training protocol that focused on barriers that hindered OPs from using the guideline, and developed solutions to overcome these barriers. Although, several more recent implementation models have been developed [4,5], we chose to use Cabana’s model [13] because it is a generic model, which is well-suited to guide barriers analyses and is still being used in various health care settings [12,29,30]. In addition, it takes into account the different stages of implementation. For example, knowledge-related barriers may be most relevant at the beginning of the implementation process; later on insight can be gained into perceived attitude-related and external barriers. In the guideline training the evolution of barriers over time can be taken into account. According to Cabana’s model, guideline adherence can be affected by three main categories of barriers: 1) knowledge-related barriers (lack of awareness/familiarity), 2) attitude-related barriers (lack of agreement, self-efficacy, outcome expectancy, and motivation), and 3) external barriers that hinder physicians from applying the guideline in practice (guideline, environmental, and patient related factors) (see Table 2).

**Table 2:** **Possible barriers to adhering to guideline recommendations in practice based on the Cabana et al. model** [13]*

**Knowledge-related barriers**	
*Lack of awareness/familiarity:*	OPs may be unaware of the (exact) content of the guideline recommendation
**Attitude-related barriers**	
*Lack of agreement:*	OPs may disagree with the guideline recommendation due to a perceived lack or inadequate interpretation of evidence or due to a lack of applicability of the recommendations in general and more specifically to individual patients
*Lack of self-efficacy:*	OPs may believe that they cannot perform the guideline recommendation because they lack appropriate training or experience
*Lack of outcome expectancy:*	OPs may believe that even if they can perform the recommendation it will not affect patient outcomes
*Inertia of previous practice/lack of motivation:*	OPs may not follow recommendations because of the difficulties of changing habits or old routines, or a lack of motivation
**External barriers**	
*Patient factors:*	OPs may be unable to reconcile patient preferences and demands with the guideline recommendations, or they may believe that patients are unable to perform the necessary actions
*Guideline recommendation factors:*	OPs may believe that the guideline recommendations are unclear or ambiguous, incomplete, or too complex
*Environmental factors:*	OPs may be unable to overcome barriers in their practice environments, such as a lack of time (time pressure), a lack of resources/materials, a lack of reimbursement, and organizational constraints within their own practice, in other organizations (e.g., out-of-hours services and pharmacies), or between organizations (e.g., cooperation and arrangements with medical specialists and GPs)

*Adapted version from Lugtenberg et al. [29]; OP = occupational physician.

To explore the perceived barriers of OPs, and to find suitable solutions to overcome these barriers, we used a ‘Plan-Do-Check-Act’ cycle. The PDCA cycle follows a learning approach to adopt changes aimed at improvement. It also provides flexibility to adapt the changes according to feedback, which helps to ensure that fit-to-purpose solutions are developed [27]. As a pragmatic scientific method, the PDCA cycle can be used in complex systems as a small-scale, iterative approach to implement, test, and improve interventions.

The focus on perceived barriers (i.e. the Cabana model) in combination with a PDCA approach formed the basis of the training on the guideline ‘MHP’ (see Table 3).

**Table 3:** **Intended structure of the guideline training ‘Mental Health Problems’**

Structure (Plan-Do-Check-Act)	*Explanation*
Stepwise discussion of the guideline content (Plan1)	In each meeting, the recommendations of part of the guideline are discussed
Barrier analysis: knowledge, attitude, and external barriers (Plan2)	Identify individual and group barriers that hinder OPs from using the guideline by discussing guideline recommendations (a different part of the guideline in each meeting)
Discussion of possible solutions for specific barriers (Plan3)	OPs discuss how specific barriers can be overcome by suggesting solutions to apply in practice
Action plan (Plan4)	OPs draw up an action plan of how to implement these solutions in their daily practice, and agree on learning objectives and ‘homework’ assignments
Practice of suggested solutions (Do)	OPs test the suggested solutions to experience how and if these would help in applying the guideline recommendation
Evaluation of experiences (Check)	OPs’ experiences with the suggested solutions are evaluated to decide what did work and what did not work for performing the guideline recommendation
Adjustment of solutions if necessary (Act)	If necessary, the solutions are adjusted according to what OPs experience in practice

OP = occupational physician.

### Protocol of the training on the ‘Mental Health Problems’ guideline

The protocol of the guideline training ‘MHP’ is described in Table 4. The guideline training consisted of eight meetings of 2 hours each spread out over a 1-year period. Small interactive groups of four to six OPs were utilized to stimulate involvement and in-depth discussion about perceived barriers and potential effective solutions. Through this peer-group learning approach, OPs interacted with other OPs and learned from each others’ experiences, knowledge, and skills to attain a common goal (i.e., make optimal use of the MHP guideline) [31]. A trainer (MJ) guided the groups by structuring the meetings, facilitating the discussion, and monitoring the progress of the groups and their training. On request, the trainer also provided course materials and tools that could help OPs overcome specific barriers.

**Table 4:** **Protocol of the guideline training**

Goals of the meetings	PDCA	Intended approach to achieve the goals
Meeting 1: Introduction of group members, guideline training, and the guideline	n/a	1. Introductory game to get to know peers and the trainer
	n/a	2. Discuss the aim and structure of the guideline training, and explain the rules of the training (confidential setting, respecting each others’ opinions, constructive feedback, role of the trainer, and role of peers)
	n/a	3. Discuss OPs’ expectations of the guideline training
	n/a	4. Briefly discuss guideline content, its weaknesses, and its strengths
Meeting 2: Discuss the *‘*Preconditions’ of the guideline and recommendations of chapter 1 ‘Problem orientation’; identify related barriers, discuss specific solutions, and draw up an action plan	n/a	1. Evaluate the previous meeting: OPs’ experiences
	n/a	2. Trainer explains the framework of Cabana et al. [13]
	P1-2	3. Discuss ‘Preconditions’ to using the guideline: the trainer asks OPs about their knowledge, attitude, and use of the guideline in practice, as well as the reasons for not using it and what would help them use it in practice
	P2-3	4. Group assignment on ‘Problem orientation’: discuss in pairs the questions to be asked to inventory patients’ problems; group discussion and check agreement with guideline recommendation; discuss what would facilitate or hinder using this recommendation; and discuss what would help facilitate use in practice
	P4, D	5. Action plan: group discussion on what the most important barriers and feasible solutions are; formulate collective learning objectives, strategies, and homework assignments
Meeting 3: Discuss guideline recommendations of chapter 1 ‘Diagnosis’; identify related barriers, discuss specific solutions, and draw up an action plan	C, A	1. Evaluate action plan: were solutions tested? What were the implementation facilitators and barriers? Discuss new solutions for barriers
	P1	2. Trainer explains key recommendations related to ‘Diagnosis’
	P2-3	3. Case discussion: one OP introduces a case, and other OPs ask questions and set diagnosis, check agreement with guideline recommendation, and discuss facilitators and barriers for use in practice
	P4, D	4. Action plan: group discussion on what the most important barriers and feasible solutions are; formulate collective learning objectives, strategies, and homework assignments
Meeting 4: Barrier analysis, and discuss solutions for guideline recommendations of chapter 2 ‘Interventions focusing on patients*’* and *‘*Process-based approach’	C, A	1. Evaluate action plan: were solutions tested? What were the implementation facilitators and barriers? Discuss new solutions for barriers
	P1	2. Trainer explains key recommendations related to ‘Interventions focusing on patients’ and ‘Process-based approach’
	P2-3	3. Case discussion: discuss possible interventions for a case, practice interventions using the case description, and check agreement with the guideline recommendations
	P4, D	4. Action plan: group discussion on what the most important barriers and feasible solutions are; formulate collective learning objectives, strategies, and homework assignments
Meeting 5: Barrier analysis, and discuss solutions for guideline recommendations of chapter 2 ‘Interventions focusing on work environment’	C, A	1. Evaluate action plan: were solutions tested? What were the implementation facilitators and barriers? Discuss new solutions for barriers
	P1	2. Trainer explains key recommendations related to ‘Interventions focusing on work environment’
	P2-3	3. Intervention tools: discussion of tools associated with the guideline; discuss knowledge, attitude, and use of the guideline in practice, as well as the reasons for not using it and what would help with use in practice
	P4, D	4. Action plan: group discussion on what the most important barriers and feasible solutions are; formulate collective learning objectives, strategies, and homework assignments
Meeting 6: Barrier analysis, and discuss solutions for guideline recommendations of chapters 3 and 4 ‘Relapse prevention, evaluation, and closure’	C, A	1. Evaluate action plan: were solutions tested? What were the implementation facilitators and barriers? Discuss new solutions for barriers
	P1	2. Trainer explains key recommendations related to ‘Relapse prevention, evaluation, and closure’
	P2-3	3. Case evaluation: OPs check each others’ cases, give feedback, and discuss agreement with guideline content
	P4, D	4. Action plan: group discussion on what the most important barriers and feasible solutions are; formulate collective learning objectives, strategies, and homework assignments
Meeting 7: Barrier analysis, and discuss solutions for guideline element ‘Process-based approach’	C, A	1. Evaluate action plan: were solutions tested? What were the implementation facilitators and barriers? Discuss new solutions for barriers
	P1-3	2. Training topics and methods adjusted to the needs of the group
	P4, D	3. Action plan: group discussion on what the most important barriers and feasible solutions are; formulate collective learning objectives, strategies, and homework assignments
Meeting 8: (Process) evaluation of the meetings	C	1. Evaluate action plan: were solutions tested? What were the implementation facilitators and barriers?
	C	2. Evaluate guideline training: OPs’ experiences of guideline training and assurance of what has been learned

Goals of the meetings, related elements of the Plan-Do-Check-Act cycle and intended approach to achieve the goals.

OP = occupational physician; P = Plan; D = Do; C = Check; A = Act; n/a = not applicable.

The training had a PDCA structure in which the content of the MHP guideline was discussed step-by-step following the chapters of the guideline (see Table 3). In the first meeting the trainer introduced herself and the participants, explained her role, and emphasized her independence towards the guideline. After providing information about the structure of the training and the role of the participants within the confidential setting the formal training started with an introduction to the guideline and general experiences with the guideline. In each subsequent meeting the PDCA structure was used; the trainer began by introducing a guideline recommendation (Plan 1 stage) and asking the OPs to discuss what hindered them from using this specific guideline recommendation in practice (i.e., barrier analysis using the Cabana model) (Plan 2 stage). Then the OPs discussed what was needed to address the perceived barriers, taking into account the context of their daily practice (Plan 3 stage). Finally, the OPs drew up a joint action plan of how to implement these solutions (Plan 4 stage). In between the meetings, the OPs tested the solutions to experience how and if these would help in applying the guideline recommendation (Do stage). In the next meeting, OPs discussed their experiences (Check stage), and, if necessary, the solutions were adjusted (Act stage); this was followed by a new plan, do, check and act stage. This PDCA cycle was repeated in subsequent meetings for all the guideline recommendations.

### Participants

The guideline training ‘MHP’ was developed as part of a larger randomized controlled trail (RCT), which aimed to explore if sick leave duration due to common mental disorders can be reduced by improving occupational health care (Trial registration: ISRCTN86605310) [32]. For this trial, OPs who were employed at a large occupational health service (OHS) in the southern part of the Netherlands were invited to participate between October 2010 and January 2011. After giving their consent, OPs were randomized to the intervention or control group. The OPs in the intervention group received the guideline training ‘MHP’ which aims at guideline-based care. OPs in the control group did not receive additional training and performed care-as-usual. OPs participated on a voluntary basis and received educational credits after completing the training. For the purpose of this feasibility study, data from the intervention group (the OPs whom received the guideline training ‘MHP’) were used. The results on the effectiveness of guideline-based care on workers’ return to work compared to care-as-usual will be described elsewhere. The research protocol of the larger RCT has been published by van Beurden and colleagues [32]. Approval was obtained from the Medical Research Ethics Committee of St. Elisabeth Hospital in Tilburg.

### Procedure and measures

To explore if the guideline training was conducted as planned, we evaluated how the training protocol (including the PDCA approach) (see Tables 3 and 4) was carried out during the training meetings. All training meetings were audio taped with the OPs’ consent, transcribed verbatim, and analyzed. Additional documents (e.g., action plan documents and the trainer’s logbook) were used to gain insight into how the training was conducted.

To enable the exploration of OPs’ experiences, the OPs answered two open-ended questions during the final training meeting on what they had learned during the training year, and were asked if they had any suggestions for improving the training. In addition, OPs rated the perceived effect of the training on their own guideline adherence on a 4-point scale ranging from 1 (no effect) to 4 (strong effect). The training components that were rated include: ‘small learning groups’, ‘eight training meetings spread over one year’, ‘focus on barriers and solutions to apply in practice’, ‘Repetition of the course material’, ‘stepwise discussion of the guideline content’, ‘PDCA structure’, ‘training topics/methods are adjusted to the needs of the group’.

For the second research question of this study—that is, the assessment of the impact of the guideline training on perceived barriers—a questionnaire based on the model of Cabana et al. [13] was filled out before and after the guideline training [29]. This questionnaire assessed participants’ knowledge, attitude, and external barriers (Table 2) by means of statements. One statement concerned the self-reported extent to which OPs adhered to the guideline (perceived adherence). A 5-point Likert scale was used to rate the extent of agreement with the statements, which ranged from 1 (strongly disagree) to 5 (strongly agree). Actual knowledge of the guideline content was assessed by a knowledge test containing 15 statements (response categories: right/wrong/don’t know) that represented the key recommendations of the guideline. One open-ended knowledge question was included to summarize the essence of the guideline. The essence of the guideline included 1) evaluation of the recovery process of the worker, 2) activating approach used by the OP, 3) identification of stagnation of the recovery process, and 4) OP acts as a process facilitator. Scoring criteria were developed based on the four essential elements of the guideline; two researchers (MJ and JvdK) independently formulated criteria, discussed disagreements, pilot tested the scoring criteria, and agreed on the final scoring criteria. Two researchers independently scored the answers on a 4-point scale ranging from 0 (very poor knowledge) to 3 (excellent knowledge).

Participants’ characteristics, such as age, education, and years of work experience, were gathered via a questionnaire at the start of the training, and were descriptively analyzed upon completion of the program. Data on the attendance of the meetings were collected during the training period.

### Data analysis

To evaluate if the guideline training was conducted as planned, the transcripts of the training meetings were briefly reviewed to get an initial impression of how the guideline training was conducted and how the PDCA approach had been utilized. Then, a detailed analysis was conducted and text fragments illustrating the PDCA were coded and bundled as Plan, Do, Check, or Act stage. Subsequently, multiple PDCA cycles were identified and coded to illustrate how lessons from one cycle were linked to the following cycle. Finally, the content of the text fragments was compared to the content of the ‘Action Plan’ documents in which OPs drew up their goals and suggested solutions (Plan phase) for each meeting [33]. The software program MaxQDA 11.0 was used for the above analyses, and results were further analyzed descriptively.

Self-reported information from the open-ended questions regarding OPs’ experiences was explored and similar concepts were grouped together. The number of categories was reduced and text fragments were bundled. For perceived effectiveness, the frequencies and percentages of responses to the statements (which training components were highly effective on guideline adherence) were examined.

For the second research question, descriptive statistics were used to designate knowledge, attitudes, and external barriers. We recoded the scores 1 and 2 (strongly/somewhat disagree) indicating disagreement, the score 3 indicating a neutral attitude, and the scores 4 and 5 (agree/strongly agree) indicating agreement. To assess actual knowledge of the guideline content, the percentage of correctly answered questions before and after the training were compared. The scores on the open-ended knowledge question were dichotomized with the scores 0 and 1 indicating insufficient knowledge of the essence of the guideline, and the scores 2 and 3 indicating sufficient knowledge. To test differences before and after the guideline training on knowledge, attitude, and perceived external barriers, we performed nonparametric tests for paired samples.

## Results

### Participants

From 155 eligible OPs, 66 participated in the larger study: 34 OPs were randomized into the control group and 32 received the guideline training. Of the remainder, 46 OPs did not respond and 43 OPs chose not to participate, of which 34 (79%) were male and the mean age was 54 years (SD = 7.1; age was based on n = 29). The main reasons for nonparticipation were lack of time (n = 18), and upcoming retirement or resignation (n = 10).

The 32 OPs who received the guideline training were divided into six groups based on their geographical work location. Groups consisted of four, five, or six OPs. One OP decided not to participate before the training started due to time constraints. Of the remaining 31 OPs, the mean age was 53 years (SD = 4.3) and 17 (55%) were male. On average, the OPs had 21 years (SD = 7.1) of experience working as an OP and were working 33 hours a week (SD = 5.6); 28 OPs (90%) had previously been educated in the MHP guideline through continuing medical education.

In consultation with the OPs, the eight meetings were scheduled over the course of a year with 3 to 6 weeks between the meetings. All OPs attended eight meetings. On six occasions an OP was not able to attend a meeting of their own group and joined another group for that particular training meeting. The duration of the meetings ranged between 112 and 157 minutes.

### Feasibility of the guideline training in practice

Overall, the training protocol was carried out as planned. During the training period, iterative PDCA cycles were conducted across different topics related to the guideline recommendations in all six groups. The PDCA provided a continuous process from exploring the rationale of a guideline recommendation, to finding and testing solutions, discovering new barriers, and finding better solutions to adhere to the recommendation. The process started with a discussion of a guideline recommendation in the second training meeting. Facilitated by the trainer, the group members engaged in a discussion about the meaning, usefulness, and reasons for using or not using the recommendation in practice. This process also helped group members identify barriers related to knowledge and attitude as well as external barriers (Plan stage). As knowledge and understanding of the guideline recommendations was often lacking, the trainer disseminated information to the group and facilitated peer discussion about the rationale of the specific recommendation. Through this process, OPs overcame important knowledge barriers in the Plan stage, leading to more in-depth discussion about attitude-related and external barriers. Also in the Plan stage, practical solutions for barriers were discussed and OPs agreed on learning goals and defined action plans to achieve the goals. In each meeting, these commonly formulated goals and ‘homework assignments’ were summarized in an ‘Action Plan’ document which the trainer sent to the OPs in the group. Not all OPs managed to test the suggested solutions between the meetings (Do stage). The reasons for not testing solutions, which included lack of time or resources and lack of motivation or confidence, were discussed in the next meeting (Check stage). Also, positive experiences with solutions were shared and discussed with the group members. During these discussions, OPs identified new barriers and suggested new solutions or adjustments to improve adherence to the guideline recommendation (Act stage). Therefore, the Plan stage of the next PDCA cycle started at this point, profiting from the experience from the previous cycle (i.e., the Act and Plan stages merged). To illustrate how OPs were engaged in the implementation of a specific guideline recommendation an example is presented in Additional file 1.

The trainer provided structure for the meetings and facilitated discussion by creating a confidential setting, giving constructive feedback to OPs, and respecting all opinions. This resulted in in-depth discussion on the topics that the OPs themselves found relevant for their context. The trainer also provided information between groups, such as educational materials, tools, and tips on suggested solutions. Moreover, the trainer stimulated co-creation of practical tools by transferring information from one group to another.

### Occupational physicians’ opinions of and experiences with the guideline training

#### Perceived effectiveness of training elements

Of 31 OPs, 28 (90%) perceived that ‘small groups’ and ‘eight training meetings spread over one year’ strongly contributed to higher guideline adherence. ‘Repetition of the course material’ and the ‘focus on barriers and solutions to apply in practice’ were second and third most mentioned (84% and 73% respectively). More than half of the OPs perceived ‘stepwise discussion of the guideline content’ (61%) and the ‘PDCA structure’ (52%) as strongly effective for guideline adherence. Least mentioned was ‘training topics/methods are adjusted to the needs of the group’ (29%).

#### OPs’ experiences

In the self-reported data from the open-ended questionnaire, OPs indicated that implementation of a guideline was an intensive process which takes more than dissemination of the (content of the) guideline alone. According to OPs, the PDCA helped them change their behavior and adopt a new working routine. OPs were also aware that it would take effort to integrate the guideline fully in their work practice. OPs mentioned that the peer-group learning approach was of added value for recognizing, for example, that their peers face the same problems and difficulties, for discussing and comparing examples and cases, for learning from each other, and for sharing practical tools.

Most OPs mentioned that their knowledge of the content, recommendations, and rationale of the guideline had increased through their attendance of the guideline training. In addition, OPs had learned how to work according to a shared structure and improve their reporting in patients’ medical records. OPs indicated that they were more aware of their own actions and limitations, and the role they played in guiding patients with MHP. OPs also mentioned that they enjoyed working with patients with MHP and felt empowered to cooperate with other caregivers. Finally, OPs found that some external factors, especially time constraints, were persistent barriers to adherence to the guideline. Consultation time with the patient was too short and a heavy work load made it difficult to put suggested solutions into practice and discuss problems or topics with their peers.

#### Suggested improvements

When asked for suggestions to improve the training, OPs indicated that follow-up meetings should be included after the 1-year training period to maintain the results achieved (n = 7). One group continued the meetings (without the trainer) quarterly to discuss guideline topics, give feedback to case reports, and share good practices. Other suggested improvements were related to the planning of the training meetings (i.e., leave more time between the meetings to test solutions/do homework [n = 4]), and to the facilities of the training (i.e., improve catering during the meetings [n = 3]). In addition, OPs suggested discussing more individual case reports (n = 3) and developing and sharing more practical tools with their peers (n = 5). Furthermore, four OPs suggested continuing this training concept for other OPs and for guidelines on other topics. Eleven OPs indicated that they had no suggestions for improvement.

### Impact on knowledge, attitude, and perceived external barriers

Table 5 presents the percentage of OPs who mentioned specific barriers related to the guideline recommendations before and after the training. Before the training, 16.1% and 35.5% of the OPs perceived barriers related to knowledge and self-efficacy respectively; afterward, none of the OPs perceived these barriers (p = .03 and p < .01 respectively). Inertia of previous practice/lack of motivation decreased from 51.6% to 25.8% after the training (p = .04), and lack of outcome expectancy was not perceived as a barrier before or after the training. External barriers related to patient ability and behavior (from 54.8% to 33.3%) and OPs’ lack of time (from 46.7% to 48.4%) remained prevalent after the training. Self-reported guideline adherence rose from 48.8% to 96.8% (p < .01) after the training.

**Table 5:** **Impact on knowledge, attitude and perceived external barriers**

	t0		t1		p-value ^ a ^
	N	freq (%)	N	freq (%)	
**Knowledge-related barriers**					
Lack of awareness/familiarity	31	5 (16.1%)	31	0 (0%)	.03
**Attitude-related barriers**					
Lack of agreement					
Lack of evidence	31	2 (6.5%)	31	0 (0%)	.16
Lack of applicability in general	31	15 (48.4%)	31	8 (25.8%)	.09
Lack of applicability to individual patients	31	14 (45.2%)	31	5 (16.1%)	.01
Lack of self-efficacy	31	11 (35.5%)	31	0 (0%)	<.01
Lack of outcome expectancy	31	0 (0%)	31	0 (0%)	1
Inertia of previous practice/lack of motivation	31	16 (51.6%)	31	8 (25.8%)	.04
**External barriers**					
Patient factors					
Patient preferences/demands	31	6 (19.4%)	29	1 (3.4%)	.22
Patient ability and behavior	31	17 (54.8%)	30	10 (33.3%)	.09
Guideline factors					
Guideline recommendation factors	31	5 (16.1%)	31	2 (6.5%)	.38
Environmental factors					
Time pressure/lack of time	30	14 (46.7%)	31	15 (48.4%)	1
Lack of resources/materials	29	3 (10.3%)	30	2 (6.7%)	.63
Organizational constraints	31	10 (32.3%)	31	3 (9.7%)	.07
Lack of reimbursement	31	7 (22.6%)	29	3 (10.3%)	.29

Mean percentage of occupational physicians who agree with the perceived barriers in adhering to the guideline ‘Mental Health Problems’ before (t0) and after (t1) the guideline training.

^a^McNemar test for paired samples.

Actual knowledge examined by the knowledge test showed that before the training 9.7% of the OPs had correctly answered 75% (or more) of the questions, versus 61.3% afterward (p < .01). Knowledge of the essence of the guideline increased nonsignificantly from 35.5% to 48.8% of the OPs (p = .39).

## Discussion

The results of this study suggest that the training in the MHP guideline is a feasible and useful implementation strategy for OPs. The strategy was carried out as planned: perceived barriers related to knowledge, attitude, and external factors that hinder OPs from using the guideline were identified and tailored interventions to overcome these barriers were implemented. Several PDCA cycles were conducted and lessons from one cycle were linked to the following cycle (i.e., adjustments to the interventions were made and tested again). In general, participating OPs had positive experiences with the guideline training. OPs’ knowledge of the guideline content increased during the training, and they also developed a more positive attitude towards the guideline. They were more aware of their own working patterns and points of attention and recognized that focusing on barriers and solutions could help them change their behavior and adopt a new working style. In addition, OPs perceived that the small peer-group learning approach and the repetition of the guideline content with meetings spread over a 1-year period contributed the most to a higher perceived guideline adherence. After the guideline training OPs perceived no knowledge barriers and were more confident and motivated to work according to the guideline than they were before the training. They still perceived time constraints in adhering optimally to the guideline.

Based on our results, a peer-group learning training with focus on perceived barriers using a PDCA structure seems to be a feasible and powerful approach to conduct a tailored implementation strategy because the target users themselves develop the solutions to overcome perceived barriers. In addition, the peer-group learning approach was highly appreciated by the OPs, as this not only created a sense of openness, it also inspired and empowered them. It gave OPs the opportunity to work together on the same goal. Enhancing the exchange of knowledge through the actively involved physicians, covering relevant clinical topics, and facilitating the acquisition of knowledge and competence simultaneously are valued elements of peer-group learning [34]. Previous studies have shown that peer-group learning activates the preknowledge of participants, leads to high-quality learning groups, and can impart sustainable knowledge and performance change [34,35]. Learning from peers in small group interactive education sessions to improve guideline adherence was also found to be highly valued by other practitioners, such as general physicians [36].

The adoption of the model of Cabana et al. [13] as a framework proved useful in understanding the barriers to implementation. In previous studies using tailored interventions, researchers mostly failed to develop and test interventions to overcome barriers perceived by physicians [16,37]. The use of the model of Cabana et al. [13] and the PDCA approach allowed the interventions to be developed by the physicians themselves, but also allowed them to test the interventions in practice and discover new barriers of which they had not been aware. Therefore, the focus on perceived barriers among the target group in combination with the PDCA approach seems to be a promising strategy to overcome identified barriers with tailor-made implementation interventions. As the strategy has a formal training structure existing of 8 sessions and can be easily adapted to another context, it is also suitable for continuing medical education purposes.

When comparing actual knowledge with perceived knowledge of the guideline, discrepancies were found that suggest that OPs overestimated their knowledge of the guideline. Before the training, only five OPs perceived lack of knowledge. But as was shown by the knowledge test, more than 90% of OPs did not correctly answer three quarters (75%) of the knowledge questions. In addition, during the training a lack of understanding of the recommendations was one of the primary barriers keeping OPs from using the guideline correctly. This suggests that OPs either found it difficult to assess their own barriers correctly, or felt reluctant to reveal their limitations (e.g., lack of knowledge) before the training started [38]. This finding confirms that it is important for barrier analyses to be performed on the level of specific recommendations [14,16]. Actual knowledge, measured with yes/no statements reflecting guideline recommendation, improved significantly after the training. But we found no improvement in knowledge of the essence of the guideline. As the guideline training mainly focused on OP’s understanding specific guideline recommendations, we expected most improvements to be found on the level of recommendations.

Time constraints remained the most prevalent perceived barrier after the training period. This was shown in the results from the questionnaire (before–after), it was also reported in the open-ended questions, and it was a recurrent topic during the training meetings. In addition, other external barriers, such as patient ability and organizational constraints did not decrease much after the training. External constraints might be too extensive and complex to be changed by a professional-directed intervention as our implementation strategy. Especially in the occupational health care setting, where the OP has to deal with national legislation, their own organization (OHS), the worker’s work environment, health care providers, care givers, and the interaction between these stakeholders. Some external constraints might be overcome by, for example empowering professionals to change their behavior and influence their environment. However, to overcome external constraints, interventions that focus directly on the organization, such as feedback systems or computerized decision aids, may be needed, and should also involve all relevant stakeholders who are committed to implementing the interventions [39-41].

Research on tailored implementation strategies specifically for occupational mental health care is scarce. A multifaceted intervention for the Dutch depression guideline for insurance physicians was found to be effective in a controlled setting [42]. In primary care, a tailored implementation strategy to improve management of anxiety and depressive disorder was found useful and may enhance guideline implementation [43]. However, conclusive evidence about the effectiveness of tailored implementation strategies is lacking, mostly because it could not be determined whether relevant barriers were identified and if they had been addressed by fit-to-purpose interventions [44]. Baker and colleagues [44] concluded that more research, such as process evaluations, is needed on how to identify and overcome barriers. Our study suggests that it is possible to identify barriers and intervene through an intensive peer-group training protocol with tailor-made interventions.

Based on this study, some adjustments to the implementation strategy could be considered. To maximize the continuity of the achieved changes, models of change [45] advise constant reminders of the desired behavior. Therefore, in future research we recommend follow-up meetings as the OPs suggested. In addition, other moderating components may influence the degree to which the guideline training was implemented, such as the (facilitating) role of the trainer [46,47]. Facilitators play an important role in assisting individuals and teams with identifying what needs to be changed and how to make these changes [48,49]. Not only should the trainer structure the meetings, facilitate the discussion, and share tools, but they should also be knowledgeable of the guideline content, communicate with participants, and build relationships between group members. As the trainer might be able to influence how well the learning groups work, we recommend selecting the trainer carefully. The role of the training should be taken into consideration when evaluating the intervention.

A strength of this study is that we used a theory-based approach and were able to implement interventions that were tailored to individual barriers to guideline compliance. In this way, OPs were able to explore what their individual needs were, find suitable solutions, and test the effectiveness of these solutions in daily practice. In addition, we used a generic method to implement a specific guideline within a specific target group. It is therefore expected that our implementation strategy is suitable for transferring to a wider range of guidelines and professionals. More research is needed to test if the current strategy is feasible and useful in different settings.

Some limitations of this study need to be mentioned. First, all OPs participated on a voluntary basis and were highly motivated to learn, which may explain their positive attitude towards the guideline training. OPs with a positive attitude towards the MHP guideline may be overrepresented in our study. This limitation is inevitable in studies addressing change in professional behavior, as willingness to change (or at least willingness to explore change) is a prerequisite to participate. In addition, interventions that ask for active participation of health professionals generally require a high degree of motivation [50,51]. Second, a bias toward social desirability may have influenced the answers concerning perceived barriers and satisfaction with the training. Third, only a small number of OPs were included in our study. Although our sample of OPs is representative of the total population of Dutch OPs in terms of basic characteristics [52], female OPs were slightly overrepresented. Also, all OPs were employed by the same occupational health service that gave participating OPs the opportunity to follow the 16-hours training. In addition, one trainer performed all of the training. These limitations indicate the need for care when generalizing the results and replicating the implementation strategy.

To improve guideline adherence, addressing perceived barriers among the target group is often considered to be a first important step [11]. After demonstrating that OPs perceived less barriers to use the MHP guideline by following the guideline training, the next step would be to examine its effect on actual guideline use objectively. Also future research should focus on the (cost) effectiveness of guideline use on clinical outcomes and, in the case of the MHP guideline, on work participation outcomes.

## Conclusion

The results of this study imply that the tailored implementation strategy for OPs contributes to the knowledge, attitude, and skills of OPs in using the MHP guideline. The focus on perceived barriers in combination with a PDCA approach seems to be a feasible strategy to translate identified barriers into a tailor-made implementation intervention, and could be a promising approach to enhance guideline adherence. It is expected that this implementation strategy is suitable for a wider range of guidelines and professionals, as it is a generic approach to overcome barriers that care professionals themselves perceive in a specific situation.

## Additional file

Additional file 1:
**Example of the implementation of a guideline recommendation by OPs participating in the guideline training.**
