# Supplementary material for: Improving occupational physicians’ adherence to a practice guideline: feasibility and impact of a tailored implementation strategy
Source: BMC Med Educ. 2015 Apr 24;15:82. doi: 10.1186/s12909-015-0364-8 (PMC4469464; doi:10.1186/s12909-015-0364-8)
Supplement: Additional file 1: — Example of the implementation of a guideline recommendation by OPs participating in the guideline training. [file 12909_2015_364_MOESM1_ESM.pdf]

Additional file 1. Example of the implementation of a guideline recommendation by OPs participating in the guideline training.

In this example a specific guideline recommendation is used namely 'use the Four Dimensional Symptom Questionnaire (4DSQ) for diagnostic purposes, for monitoring symptoms and for providing feedback to the patient and/or other caregivers'.

	GROUP DISCUSSION	PERCEIVED BARRIERS	SOLUTIONS	TEST SOLUTIONS
MEETING 1	- Dissemination of guideline recommendation and information by the trainer on the purpose of the 4DSQ<br>- Discussing the guideline recommendation in peer group	Lack of knowledge/understanding of the 4DSQ	Studying guideline text	OPs test solutions in daily practice
		Limit access paper version of the 4DSQ (not available at the work site)	Creation of an electronic 4DSQ tool → trainer disseminates electronic 4DSQ tool among OPs	
		Negative attitude towards use 4DSQ (i.e. not useful in practice)	Case discussion (i.e. next meeting cases are discussed)	
		Lack of time / not used to work with 4DSQ	Case discussion (i.e. next meeting cases are discussed)	

	GROUP DISCUSSION	PERCEIVED BARRIERS	SOLUTIONS	TEST SOLUTIONS
MEETING 2	- Discuss experiences with (peer) group members<br>- Case discussion: one OP introduces case and receives feedback from peers	*Solved barriers*: Knowledge of the purpose of 4DSQ	No further actions are needed	OPs test solutions in daily practice
		Remaining barrier: Lack of time; Lack of belief that 4DSQ is useful	Individual guidance from peers	
		New barriers: Lack of skills and confidence to use 4DSQ; Difficult to adopt new assessment strategy	Share practical tips with peers from other groups (via the trainer)	

MEETING 3	GROUP DISCUSSION	PERCEIVED BARRIERS	SOLUTIONS	TEST SOLUTIONS
	- Discuss experiences with (peer) group members<br>- Share good practices (within group and between groups)	*Solved barriers*: For most OPs the electronic 4DSQ is a feasible tool; Belief that 4DSQ is an useful tool	No further actions are needed	OPs test solutions in daily practice
		Remaining barrier: Lack of skills and confidence to use 4DSQ; Difficult to adopt new assessment strategy; Lack of time	Individual case discussion	
		New barriers: OPs make limited use of 4DSQ to monitor symptoms; Difficult to apply to suicidal patients	Use 4DSQ specifically to monitor symptoms over time	

MEETING 4	GROUP DISCUSSION	PERCEIVED BARRIERS	SOLUTIONS	TEST SOLUTIONS
	- Discuss experiences with (peer) group members<br>- Individual case discussion (provide feedback to peer's case)	*Solved barriers*: OPs are skilled and confident to use 4DSQ; OPs feel empowered when using 4DSQ (e.g. to discuss 'difficult' topics with patient, in contact with other health care providers)	No further actions are needed	OPs test solutions in daily practice
		Remaining barrier: Lack of time due to workload; takes time to fully adopt a new assessment strategy	Keep evaluating the use of 4DSQ with peers	
		New barriers: Lack of skills to interpret results from 4DSQ as monitor	Keep evaluating the use of 4DSQ with peers	
